# Histopathology and surgical outcome of symptomatic treatment-related changes after gamma knife radiosurgery in patients with brain metastases

**DOI:** 10.1038/s41598-022-06881-6

**Published:** 2022-02-22

**Authors:** Jeong-Hwa Kim, Jung-Won Choi, Doo-Sik Kong, Ho Jun Seol, Do-Hyun Nam, Jae-Wook Ryu, Sung-Tae Kim, Yeon-Lim Suh, Jung-Il Lee

**Affiliations:** 1 Department of Neurosurgery, Samsung Medical Center, Sungkyunkwan University School of Medicine, 81 Irwon-ro, Gangnam-gu, Seoul, 06351 South Korea; 2 Department of Radiology, Samsung Medical Center, Sungkyunkwan University School of Medicine, Seoul, South Korea; 3 Department of Pathology, Samsung Medical Center, Sungkyunkwan University School of Medicine, Seoul, South Korea

**Keywords:** CNS cancer, Surgical oncology, Outcomes research

## Abstract

A late-onset treatment-related changes (TRCs), which represent radiographic radiation necrosis (RN), frequently occur after stereotactic radiosurgery (SRS) for brain metastases and often need surgical treatment. This study aimed to validate the true pathology and investigate clinical implication of surgically resected TRCs on advanced magnetic resonance imaging (MRI). Retrospective analyses of 86 patients who underwent surgical resection after radiosurgery of brain metastases were performed. Fifty-four patients displayed TRCs on preoperative MRI, comprising pure RN in 19 patients (TRC-RN group) and mixed viable tumor cells in 35 patients (TRC-PD group). Thirty-two patients revealed the consistent diagnosis of progressive disease in both MRI and histopathology (PD–PD group). The TRC-PD group showed larger prescription isodose volume (9.4 cm^3^) than the TRC-RN (4.06 cm^3^, p = 0.014) group and a shorter time interval from SRS to preoperative MRI diagnosis (median 4.07 months) than the PD–PD group (median 8.77 months, p = 0.004). Progression-free survival was significantly different among the three groups (p < 0.001), but not between TRC-RN and TRC-PD (post hoc test, p = 1.00), while no difference was observed in overall survival (p = 0.067). Brain metastases featured as TRCs after SRS frequently contained viable tumor cells. However, this histologic heterogeneity had a minor impact on benign local prognosis of TRCs after surgical resection.

## Introduction

With the improvement of primary site control in solid tumors using chemotherapy and immunotherapy, management of metastatic lesions has become a major concern for patients with cancer^1,2^. Current guidelines for management of brain metastases (BM) include stereotactic radiosurgery (SRS) or radiotherapy as a key modality for local control with high efficacy^3–5^.

Given the importance of radiosurgical treatment for metastatic brain tumors, neurosurgeons and oncologists often encounter treatment-related change (TRC) when treating patients with brain metastases. TRC has the following issues in clinical practice; it is not easy to distinguish TRC from PD by current imaging modalities, and the other is that direct confirmation of the pathology is not always feasible.

Differentiating TRC from progressive disease (PD) has also been a matter of debate for managing patients with TRC, because both present similar imaging appearance in conventional magnetic resonance imaging (MRI) of enlarged, heterogeneous rim enhancement in the T1-weighted sequence^6,7^. Although several advanced imaging modalities such as perfusion or diffusion weighted, MR spectroscopy, and radiomics-oriented studies have tried to discriminate TRCs from PD, they were often lack of histologic validation and remote from clinical application^8–10^. A cancer patient with poor performance status may be unsuitable for surgical resection of medical-refractory TRC. Even if a biopsy is performed, the result may not fully cover the heterogeneous pathology of a metastatic lesion.

To the best of our knowledge, there are still no consistent standard guidelines for the radiology and pathological definition of TRC. TRC has been variously classified according to the time after irradiation, and a pathological diagnosis was attached to it in previous studies. For example, acute radiation injuries were defined as transient, reversible neurotoxic phenomena that occur within several days to weeks. In contrast, early-delayed types of TRC, also called pseudoprogression, had been considered to appear several weeks to months after radiation. Radiation necrosis (RN) is a pathology commonly paired with late-onset TRC, and differential diagnosis has been made at least 3–6 months after radiation therapy. Unlike acute radiation injury or pseudoprogression, most presented an irreversible clinical course and are often accompanied by neurological symptoms, requiring active treatment^11–14^. Previous studies revealed that symptomatic focal brain necrosis occur in up to 10% of patients who undergo gamma knife radiosurgery (GKS) for BM. The overall risk of RN leading to permanent morbidity and additional surgical resections was reported as 4.7–7.0%^4,5,15^.

From these points of view, the current management of TRC mainly focuses on the control of related symptoms. Patients with symptomatic TRC are usually treated with steroids or the vascular endothelial growth factor-A monoclonal antibody bevacizumab (Avastin) as first-line treatment^16,17^, whereas there are patients for who surgical treatment is required because symptoms are not controlled. Although it is not uncommon for resection of the TRC to be necessary, the clinical course after surgery has been rarely known^18–20^.

This study aims to determine the clinical significance of TRC by investigating of the actual pathological diagnosis of late-onset TRC, and the clinical prognosis after surgical resection. In the text, TRC included only late-onset and symptomatic TRC, which requires surgical decompression, excluding other reversible pathologies of acute radiation injury or pseudoprogression. We assumed that several histological characteristics of TRCs may be the mixture of viable tumor cells with RN rather than pure necrosis. The clinical significance of TRC was examined by comparing the clinical course of patients diagnosed to be TRC on the advanced MRI but whose tumor cell was confirmed in the pathological report, to that of patients whose both imaging- and pathological diagnoses were confirmed as metastatic progression.

## Results

### Patient classification

**Figure 1:**
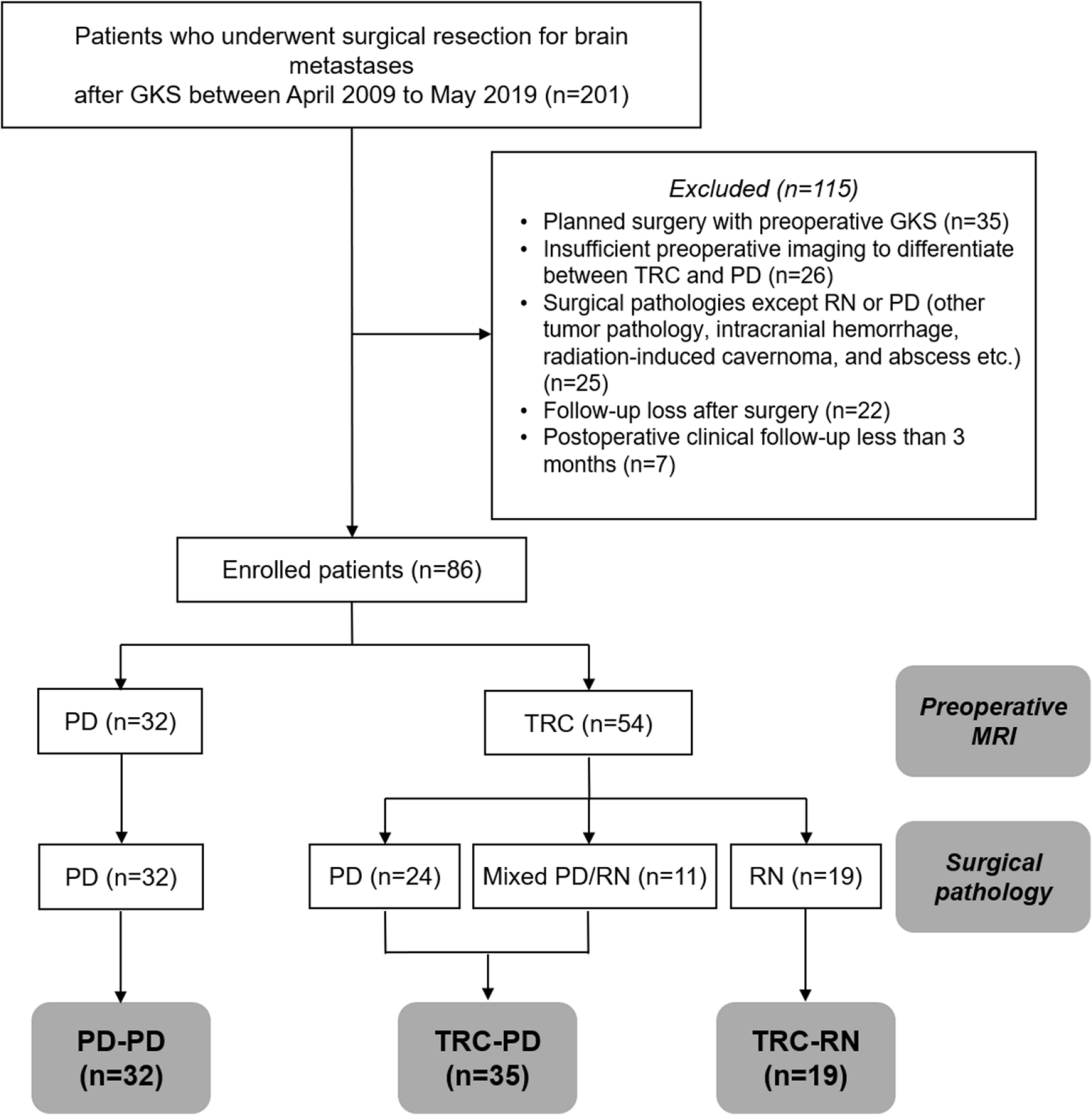
Flow chart of patient enrollment and classification. Eighty-six patients were included and classified into four groups according to preoperative MRI and surgical pathology. For surgical pathology, pure radiation necrosis alone was sorted into “radiation necrosis”. Mixed pathology, containing both metastatic tumor and necrotic cells, was classified as “metastatic cells.” No patient was assigned to the PD-RN group; therefore, the three groups TRC-RN, TRC-PD, and PD–PD were finally incorporated in this study. *MRI* magnetic resonance imaging, *PD* progressive disease, *RN* radiation necrosis, *TRC* treatment-related change.

A total of 86 patients was included in the final analysis. Based on the radiological and histopathological diagnoses, patients were classified into three groups (Fig. 1). The nomenclature of each patient group is composed as follows: preoperative radiological diagnosis (TRC or PD)—postoperative histologic diagnosis (RN or PD). As no patient who was diagnosed as PD in preoperative MRI and then RN in histologic diagnosis after surgical resection (designated as PD-RN, if such patients existed), patients were classified into three groups in final analyses; TRC-RN, TRC-PD, and PD–PD groups.

**Figure 2:**
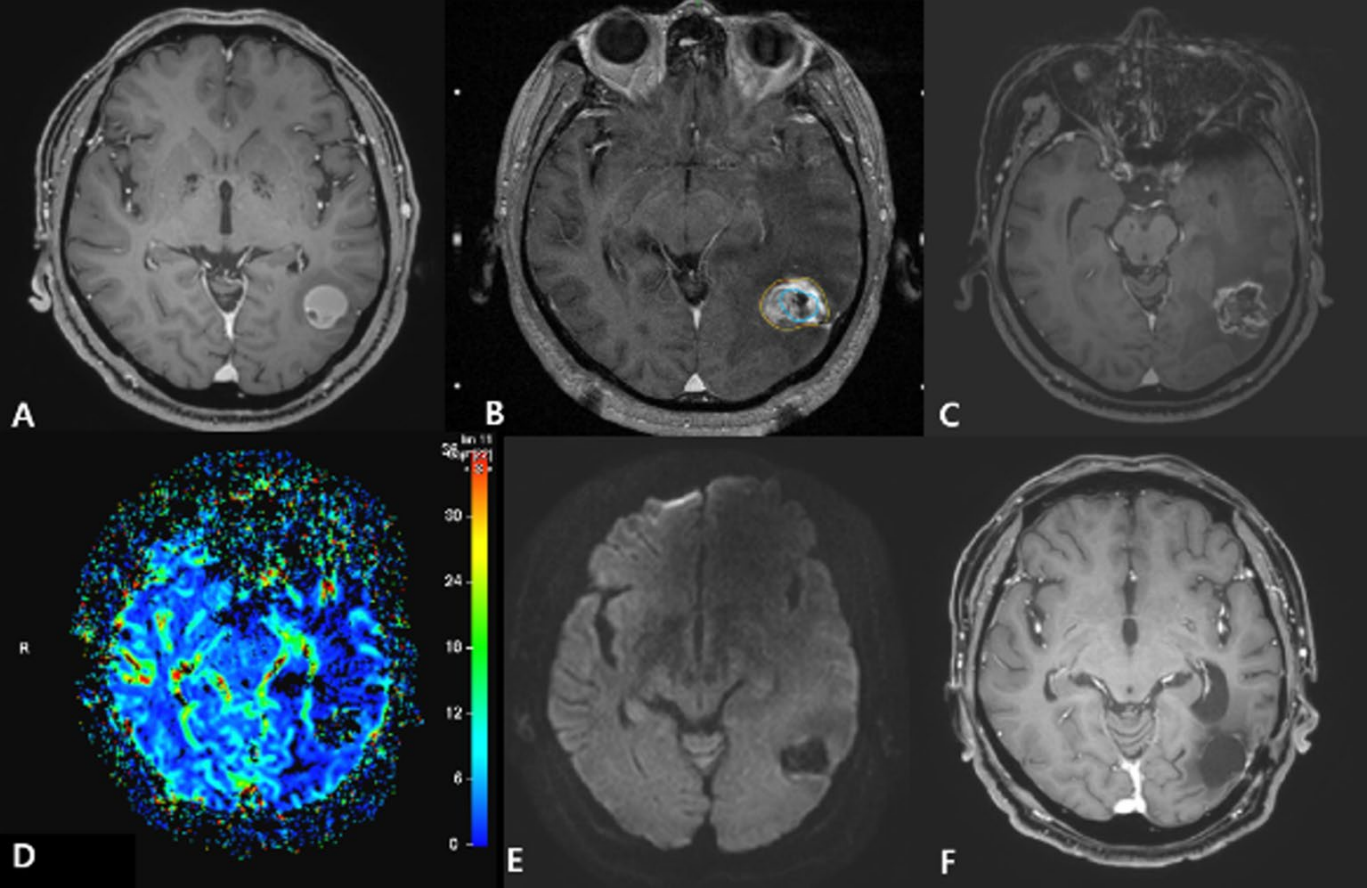
An illustrative case of TRC-PD. A 73-year-old male patient with non-small cell lung cancer was diagnosed with a 2.9 cm brain metastatic lesion in the left temporo-occipital junction (**A**). GKS with an initial prescription dose of 20 Gy at 50% IDL was performed. Due to suspicion of tumor progression at 11 months after the first session, a secondary prescription dose of 17 Gy at 50% IDL was administered (**B**, IDL of the second GKS [yellow] and previous session [blue]). At 9 months from the last GKS, the patient visited the emergency room with IICP signs, and MRI showed a lesion resembling TRC with no distinct increase in rCBV and absence of restricted diffusion (**C**–**E**). Surgical resection was conducted and the pathology was confirmed to be a pure metastatic tumor cells. Without adjuvant local therapy for the resected lesion, the patient was free of local progression for 15 months (**F**). *IDL* isodose line, *IICP* increase of intracranial pressure, *PD* progressive disease, *rCBV* relative cerebral blood volume, *TRC* treatment-related image change.

According to preoperative imaging, TRCs were diagnosed in 54 (63%) patients and PD in 32 (37%) patients. Among the patients with TRCs, the surgical pathology confirmed pure necrosis in only 19 of the 54 (35%) patients whereas the other 35 (65%) patients revealed mixed (11 of 35, 31%) or pure metastatic (24 of 35, 69%) histology, represented as the TRC-RN and TRC-PD groups, respectively. A representative case of the TRC-PD group was described in Fig. 2.

All 32 patients with MRI-diagnosed tumor progression were histopathologically confirmed as having tumor progression and included in the PD–PD group.

### Patient demographics and lesion characteristics

The preoperative patient characteristics and treatment parameters are shown in Table 1. The median age of the entire study population was 58 years (range 40–81 years). Most metastatic lesions were in the frontal (40 patients, 47%) and parietal (22 patients, 26%) regions with similar proportions in all three groups (p = 0.754).

**Table 1:** Patient demographics and treatment parameters.

	Total (n = 86)	TRC-RN (n = 19)	TRC-PD (n = 35)	PD–PD (n = 32)	p-value
Median age, year (range)	58 (40–81)	57 (40–77)	58 (40–74)	58 (41–81)	0.943
**Sex (%)**					0.879
Male	36 (41)	7 (36)	15 (42)	14 (43)	
Female	50 (58)	12 (63)	20 (57)	18 (56)	
**Primary malignancy (%)**					0.169
Lung	57 (66)	14 (73)	27 (77)	16 (50)	
Breast	15 (17)	2 (11)	5 (14)	8 (25)	
^*b*^Other	14 (16)	3 (16)	3 (9)	8 (25)	
**Multiple GKS history for surgically resected lesion (%)**					0.005
Yes	28 (33)	12 (63)	9 (26)	7 (22)	− 2.68^*a*^
No	58 (67)	7 (37)	26 (74)	25 (78)	0.007^*a*^
**Prior WBRT (%)**					0.117
Yes	18 (21)	7 (37)	7 (20)	4 (13)	
No	68 (79)	12 (63)	28 (80)	28 (87)	
**Location (%)**					0.754
Frontal	40 (46)	8 (42)	17 (49)	15 (48)	
Parietal	23 (27)	7 (37)	10 (28)	6 (19)	
Temporal	5 (6)	1 (5)	1 (3)	3 (9)	
Occipital	7 (8)	1 (5)	2 (6)	4 (12)	
Cerebellum	11 (13)	2 (11)	5 (14)	4 (12)	
Median PIV, cm^3^ (range)	6.41 (0.14–62.29)	***4.06 (0.14–34.50)***	***9.40 (1.50–40.72)***	6.80 (0.89–62.29)	0.014
Median prescription dose, Gy (range)	18 (8–25)	18 (14–25)	18 (8–22)	17 (12–23)	0.605
Single SRS (%)	70 (81)	***16 (84.2)***	25 (71)	26 (81)	0.472
Hypo-fractionated SRS (%)	16 (19)	***3 (15.8)***	10 (29)	6 (19)	
Median time interval from GKS to imaging diagnosis of TRC or PD, months (range)	6.07 (3.01–40.03)	5.43 (3.01–39.60)	***4.07 (3.40–18.13)***	***8.77 (4.10–40.03)***	0.004
**Primary malignancy control (%)**					0.413
Controlled	41 (48)	9 (47)	14 (40.0)	18 (56)	
Uncontrolled	45 (52)	10 (53)	21 (60.0)	14 (44)	
Median preoperative KPS	70 (50–90)	70 (50–90)	70 (60–80)	70 (60–90)	0.555
Median postoperative KPS	80 (40–90) < 0.001^c^	80 (40–90) 0.233^c^	80 (70–90) < 0.001^c^	80 (70–90) < 0.001^c^	0.164^d^

The overall p-value comparing the three groups is indicated on variables. If the overall p-value was less than 0.05, post hoc test was performed to compare every two groups in pairs. The variables which showed significant differences were designated in bold italics.

*GKS* gamma knife radiosurgery, *PD* progressive disease, *PIV* prescription isodose volume, *RN* radiation necrosis.

^a^z- and p-values of the Jonckheere-Terpstra test.

^b^renal cell cancer (3), colorectal cancer (4), melanoma (2), hepatocellular carcinoma (2), cholangiocarcinoma, endometrial carcinoma, thyroid cancer.

^c^In each group, the p-value for the change in KPS score before and after surgery was estimated through Wilcoxon signed rank test.

There were significant differences in multiple GKS history especially for the surgically resected lesions between the three groups (p = 0.005). A much higher percentage of patients (63%) in the TRC -RN group had a history of multiple GKS for the resected metastatic lesion, whereas nine (26%) had it in the TRC-PD group and seven (22%) in the PD–PD group (Jonckheere-Terpstra test, z =–2.68, p = 0.007). In contrast, there was no significant difference in the proportions of history of whole brain radiation therapy (p = 0.117).

The median prescription isodose volume (PIV) was 6.41 cm^3^ (range 0.14–62.29 cm^3^). Statistical differences were observed among the median PIVs of the three groups (p = 0.014), and the median PIV of the TRC-RN group (4.06 cm^3^, range 0.14–34.50 cm^3^) was smaller than that of the TRC-PD group (9.40 cm^3^, range 1.50–40.72 cm^3^) in the post hoc test (p = 0.011). Radiation doses were delivered to 50% isodose line (IDL) in all patients. The median prescription dose was 18 Gy (range 8–25 Gy) in single session radiosurgery for 72 patients. Median cumulative prescription doses were 18 Gy in two fractions for one patient, 24 Gy (range 21–30 Gy) in 3 fractions for 14 patients, and 30 Gy in 4 fractions for one patient. The proportion of patients who underwent hypofractionated radiosurgery showed no significant difference among the three groups (p = 0.472).

The median time from the date of GKS to the imaging diagnosis was 6.1 months (range 1.0–40.0 months). Upon post hoc analysis, the TRC -PD group exhibited a shorter time to imaging diagnosis than the PD–PD group (4.1 months, [range 1.0–18.1 months] vs. 8.8 months [range 1.0–40.0 months], respectively; p < 0.001).

### Surgical outcomes and salvage therapy

81 patients (94%) underwent surgical resection within 4 weeks and the other five patients within 6 weeks after the time of radiologic diagnosis. Preoperative neurological deficits were headache in 57, motor weakness in 27, nausea and vomiting in 16, seizure in 13, and aphasia in 10 patients. Total resection of the lesions was conducted in most of the patients to relieve the cerebral mass effect and promote better local control, except two patients in the PD–PD group underwent subtotal lesionectomy due to a poorly demarcated tumor related to irradiation or adherence to an anatomically critical structure such as the anterior clinoid process.

The median Karnofsky Performance Scale (KPS) score of total patients improved after surgical resection (preoperative KPS 70 (range 60–90) and postoperative 80 (range 40–90), p < 0.001). In subgroup analyses, the pathologic PD group showed significant improvement in KPS after surgery (p < 0.001 in both TRC-PD and PD–PD groups), meanwhile the TRC-RN group showed no significant change in KPS before and after surgery (p = 0.233) (Table 1). The neurological deficits improved for most patients (66, 77%) but were sustained or worse after surgery in the other 20 (23%) patients. No major intraoperative complications were observed. One patient had newly developed hemiplegia in the immediate postoperative status, a predictable complication due to the location of the tumor of the left centrum semiovale with infiltration of thalamus.

After surgical resection, the chemotherapy regimen was changed according to disease progression status in the TRC-PD and PD–PD groups. The TRC-RN group was assessed as having stable disease and maintained the regimen. Postoperative GKS for the tumor bed was applied in only two patients in PD–PD group due to subtotal removal of the PD lesion, within a month after surgery. Other patients with total removal of metastatic lesions were clinically followed-up without any adjuvant local therapy and additional radiosurgery or surgical resection was performed only if local or distant progression was confirmed in radiologic evaluation.

### Survival analysis

**Figure 3:**
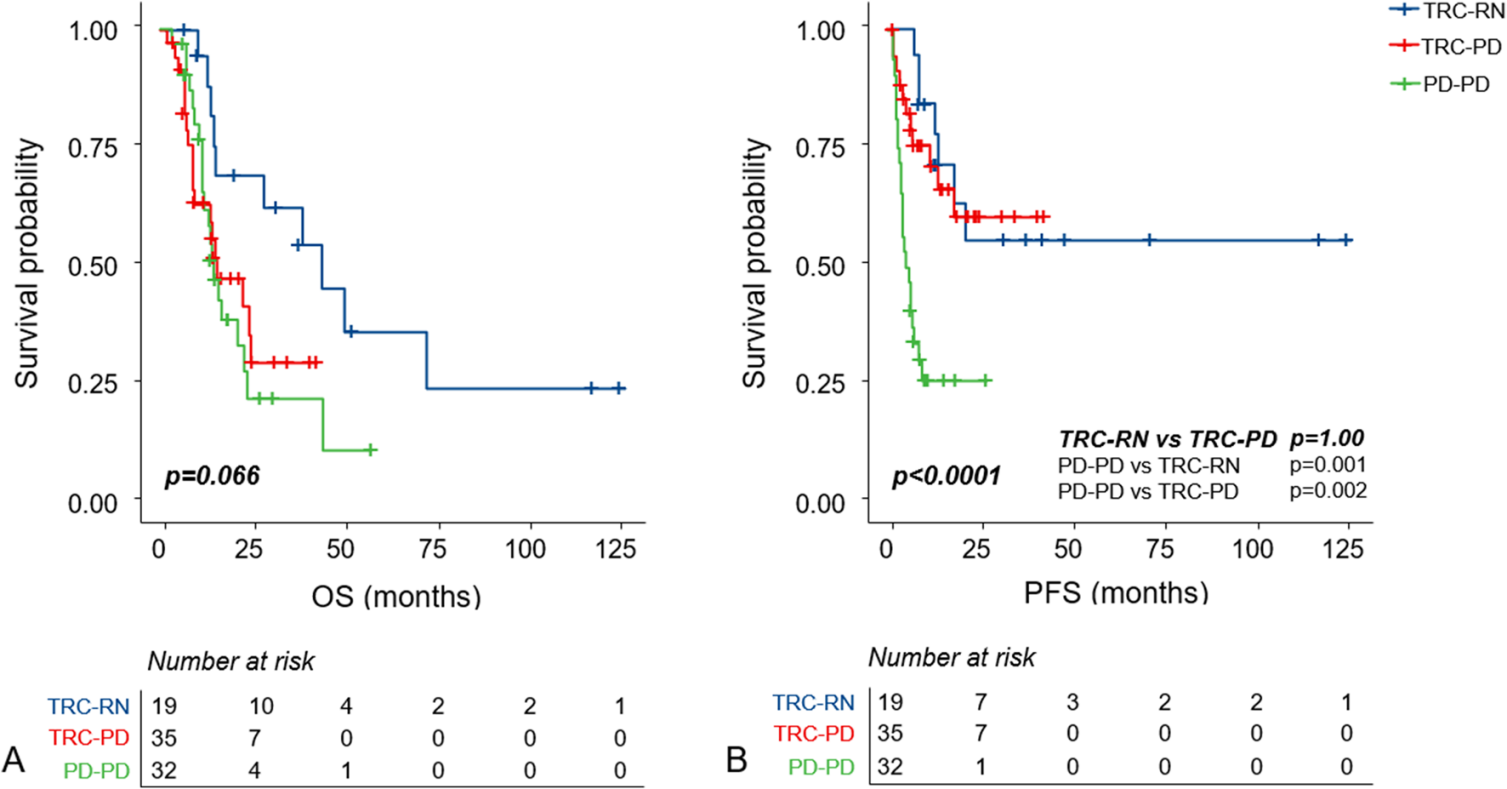
Kaplan–Meier estimate of overall survival (**A**) and progression-free survival (**B**).

Survival outcome and Kaplan Meier (KM) estimation for local recurrence and all-cause death were described in Table 2 and Fig. 3, respectively.

**Table 2:** Summary of clinical outcomes after surgical resection to GKS-treated metastases.

	Median follow-up (range)	Median OS (range)	Median PFS (range)	6-months LC	1-year LC
TRC-RN	27.7 (7.6–126.5)	43.7 (34.7–51.8)	NA*	84%	78%
TRC-PD	12.8 (2.3–43.9)	16.0 (5.1–26.9)	NA*	79%	68%
PD–PD	14.1 (3.6–58.8)	15.2 (12.2–18.2)	5.4 (2.7–8.0)	41%	26%

*LC* Local control, *NA* Not applicable, *OS* Overall survival, *PFS* Progression free survival.

*Median PFS of TRC-RN and TRC-PD were not applicable because less than 50% of patients were confirmed with progressive disease after surgical resection during the follow-up period.

Median follow-up period of total patients was 14.3 months (range 2.3–126.5 months). Forty-one patients (47%) exhibited local progression of the resected lesion; 7 (36%) in TRC-RN, 11 (47%) in TRC-PD, and 23 (71%) in PD–PD. TRC-RN group achieved the highest local control (LC) rate among the three groups at both 6-month LC (84%) and 1-year LC (78%). However, there was no significant difference in progression-free survival (PFS) between the TRC-RN group and the TRC-PD group (Fig. 3B, p = 1.00). The PD–PD group showed the highest recurrence rate (6-month LC = 41% and 1-year LC 26%) with a significantly worse PFS from other groups (Fig. 3B, PD–PD vs. TRC-RN; p = 0.001 and PD–PD vs. TRC-PD; p = 0.002).

The median overall survival (OS) for the 86 patients was 21.7 months (95% confidence interval [CI]: 12.9–30.6 months). Patients in the TRC-RN group displayed a tendency toward more prolonged survival than patients in the pathologic PD group: 1-year and 2-year survival was 82% and 75%, respectively (Table 2). KM estimation revealed a statistical trend of different OS between three group, however, significance was not reached (Fig. 3A, p = 0.067).

### Subgroup analysis of TRC-PD group

**Figure 4:**
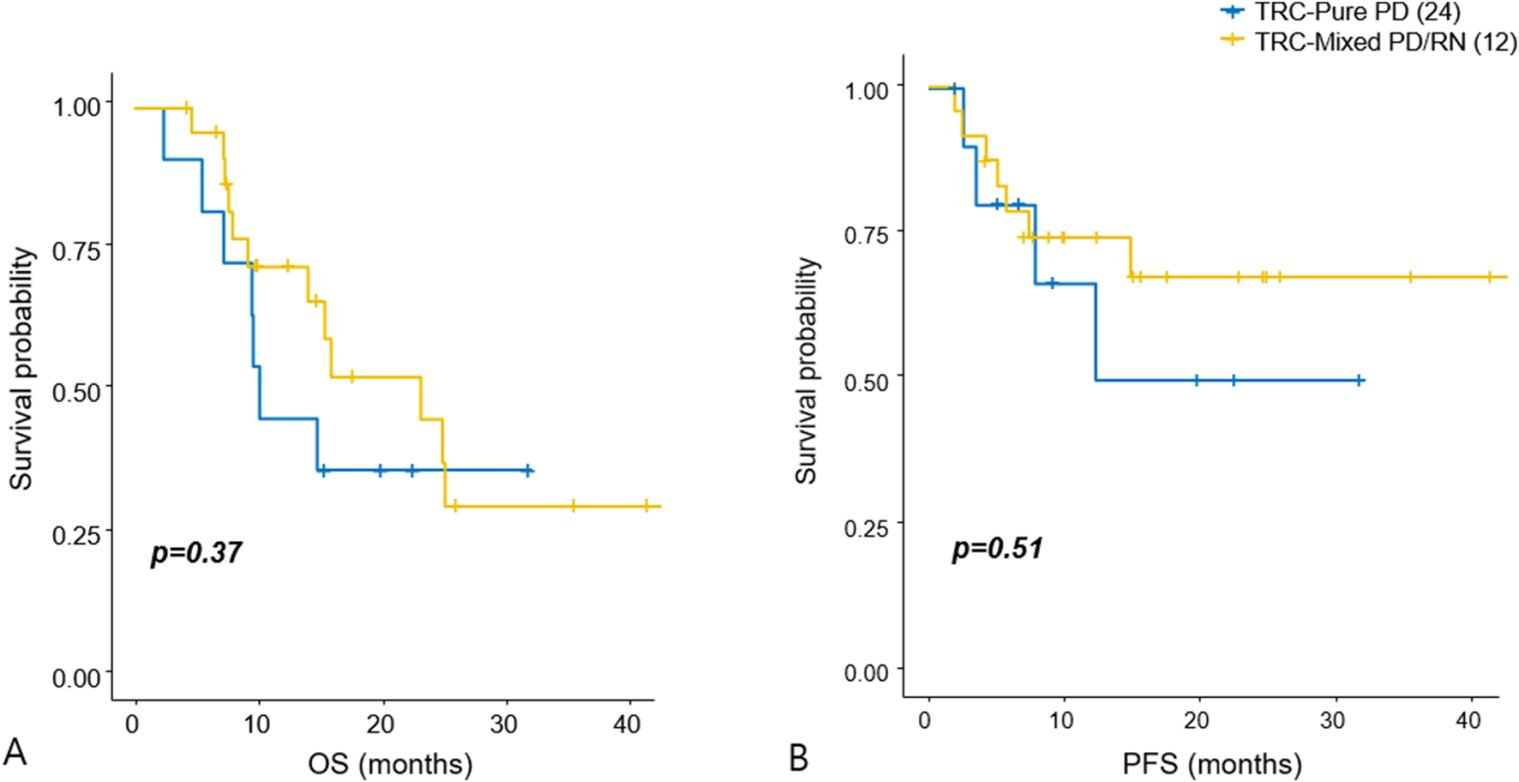
Subgroup analysis of patients with TRC-PD. Comparison of the KM curve of OS and PFS between TRC-PD patients with pure metastatic tumor cells (n = 24, **A**) and mixed pathology of radiation necrosis and progressive tumor (n = 11, **B**), respectively. *OS* overall survival, *PD* progressive disease; *PFS* progression-free survival, *RN* radiation necrosis, *TRC* treatment-related change.

For details of histopathological investigation of clinical prognosis, we further stratified the TRC-PD group according to its surgical pathology, as “TRC-Pure PD” including metastatic tumor cells alone (24 patients) and “TRC-Mixed PD/RN” including mixed pathology of radiation necrosis and viable tumor cells (11 patients). The Kaplan–Meier curve and log-rank tests revealed no significant difference between two subgroups of patients with TRC-PD either for OS or local progression (Fig. 4A,B). These results suggest that the histopathologic heterogeneity of TRCs did not have influence on the clinical prognosis of metastatic lesions after surgical resection.

### Risk factors for local recurrence and all-cause death

Table 3 presents the results of cox regression analyses using clinical variables. There was a higher risk of local recurrence in the PD–PD group, with an adjusted hazard ratio (HR) of 5.461 (95% CI: 1.857–16.061, p = 0.002) in the multivariable analysis, compared with that in the TRC-RN group, whereas no significant difference for the risk of local progression between the TRC-RN and TRC-PD groups (p = 0.535). As for the primary cancer type, the risk of recurrence was higher in the non-lung cancer group than in the lung cancer group in univariable analyses. (Overall p = 0.012, Breast cancer; unadjusted HR 2.809 [95% CI 1.317‒5.990], p = 0.008 and other types of cancer; unadjusted HR 2.292 [95% CI 1.050‒1.883], p = 0.037).

**Table 3:** Cox regression analyses for the risk of death and tumor recurrence.

	All-cause death				Local recurrence			
	Univariable analyses		Multivariable analysis		Univariable analysis		Multivariable analysis	
	unadjusted HR (95% CI)	p-value	adjusted HR (95% CI)	p-value	unadjusted HR (95% CI)	p-value	adjusted HR (95% CI)	p-value
Age, years	1.004 (0.974–1.035)	0.801			1.023 (0.990–1.057)	0.154		
PIV, mm^3^	0.992 (0.967–1.017)	0.519	1.000 (0.94–1.00)	0.221	1.007 (0.981–1.032)	0.615	1.001 (0.989–1.002)	0.441
Prescription dose, Gy	0.941 (0.852–1.040)	0.234			0.887 (0.796–0.989)	0.030	0.929 (0.799–1.081)	0.339
Time interval from GKS to Imaging diagnoses (TRC or PD), months	0.966 (0.930–1.003)	0.069	0.961 (0.921–1.003)	0.061	0.999 (0.968–1.031)	0.957	0.997 (0.956–1.041)	0.903
Preoperative KPS	0.956 (0.956–1.016)	0.341			0.966 (0.934–1.000)	0.049		
Postoperative KPS	0.981 (0.961–1.002)	0.075			0.988 (0.963–1.014)	0.369		
TRC-RN	Reference	0.757*	Reference	0.067*	Reference	< 0.001*	Reference	0.002*
TRC-PD	2.313 (1.008–5.308)	0.048	2.554 (0.957–6.818)	0.061	1.116 (0.432–2.881)	0.821	1.412 (0.474–4.204)	0.535
PD–PD	2.469 (1.100–5.543)	0.028	3.063 (1.008–6.700)	0.049	4.109 (1.725–9.788)	0.001	5.461 (1.857–16.061)	0.002
^a^Female	0.736 (0.414–1.310)	0.298			1.143 (0.604–2.162)	0.681		
**Primary malignancy**								
Lung	Reference	0.056*	Reference	0.216*	Reference	0.012*	Reference	0.305*
Breast	1.296 (0.610–2.753)	0.500	0.915 (0.419–2.071)	0.825	2.809 (1.317–5.990)	0.008	1.987 (0.830–4.760)	0.123
Other	2.305 (1.165–4.559)	0.017	1.634 (0.469–5.0689)	0.440	2.292 (1.050–5.002)	0.037	1.286 (0.535–3.090)	0.574
Multiple GKS history for surgically resected lesion^b^	0.715 (0.379–1.379)	0.300	0.986 (0.478–2.035)	0.969	0.974 (0.504–1.883)	0.938	1.274 (0.543–2.990)	0.578
Hypo-fractionated SRS^c^	0.851 (0.398–1.819)	0.676			1.247 (0.593–2.618)	0.560	0.679 (0.223–2.068)	0.496
WBRT^d^	0.890 (0.453–1.751)	0.736			0.633 (0.280–1.431)	0.272		
Uncontrolled primary malignancy^e^	2.027 (1.143–3.595)	0.016	1.743 (0.849–3.579)	0.003	1.420 (0.762–2.647)	0.269		

*The overall p-value of categorical data with more than two variables.

^a^Reference was male.

^b^Reference was no previous GKS for resected lesion.

^c^Reference was a single session GKS.

^d^Reference was no history of WBRT.

^e^Reference was controlled primary malignancy.

According to the analysis of risk factors for death, uncontrolled primary cancer significantly increased the risk of death (adjusted HR 1.743 [95% CI 0.849–3.579], p = 0.003). Except for lung cancer and breast cancer, other types of primary malignancy tended to be higher risk for all-cause death in the univariate analysis (overall p = 0.056; unadjusted HR 2.305 [95% CI 1.165–4.559], p = 0.017), but no significant association was observed in the multivariate analysis (p = 0.216). Other clinical factors were found to have no significant effect on the overall survival rate.

## Discussion

In this study, we investigated the clinical implication of TRC-PD representing patients with discrepant radiopathology and surgical histopathology. Despite heavy reliance on conventional imaging for progression guidelines and attention to subtleties in appearance with postcontrast T1 and FLAIR imaging, the sensitivity and specificity of standard MRI for detection of progression is low^7,21,22^. Recently, a meta-analysis was conducted by Chuang et al. to investigate the utility of PWI and magnetic resonance spectroscopy (MRS) for differentiating recurrent tumors from necrosis in patients with various brain tumors^10^. Of the 397 patients in 13 studies, 95 patients had BM. The meta-analysis revealed that the rCBV derived from PWI, as well as MRS metabolite ratios in contrast-enhancing lesions, were significantly different in local progression compared with lesions resulting from radiation-related changes. Previous studies also reported variable diagnostic performance of PWI in terms of sensitivity (range 56–100%), specificity (range 68–100%), and relative CBV threshold (range 1.52–2.14)^7,23^. Although many previous studies have tried a quantitative approach to PWI, the universal threshold for differentiating the tumor progression is not yet established and has low reproducibility. A recent survey indicated that approximately 50% of surveyed institutions do not process quantitative parameter maps due to high clinical cost-to-benefit ratio^24^. MRS appeared to provide high specificity for detecting tumor recurrence (almost 100%). However, the relatively low sensitivity (range 33–50%) and limited application for small-sized lesions (< 2 cm^3^) are its two major drawbacks^7,25^. The potential benefit of amino acid positron emission tomography for differentiating pseudoprogression or RN from true disease progression following checkpoint inhibitor treatment of BM has been suggested, although the evidence is preliminary^26^. In our study, most TRC or PD lesions, which worsened neurological deficits, were usually large with a median volume of 6.41 cm^3^ (range 0.14–62.29 cm^3^). Thus, we primarily utilized only PWI and DWI plus standard MRI protocols for preoperative imaging to achieve diagnostic accuracy and time–cost benefit in terms of determining urgent surgical resection.

The patients in the TRC-PD group had a larger PIV (median 9.40 cm^3^) than patients in the TRC-RN group (median 4.06 cm^3^), with no differences in the median prescription dose of 18 Gy (range 14–25 Gy in TRC-RN and 8–22 Gy in TRC-PD). On deriving the median diameter from the median PIV in our study, the median target size values for the TRC-RN and TRC-PD groups were 15.95 mm and 21.1 mm, respectively. For targets in this range, a marginal dose of 21–24 Gy, or at least > 18 Gy, has been suggested for local control according to previous studies^27–29^. Additionally, Amsbaugh et al. demonstrated that a unit increase in the maximum dose (Gy) per target size (mm) was associated with a decreased local failure in SRS for BM, with the requirement for a higher prescription dose being proportional to the target size^21^. This may explain the histologic local failure of TRC-PD compared with TRC-RN under the same marginal dose.

The period from the last GKS date to the time of MRI diagnosis of TRC or PD was significantly shorter in the TRC-PD group (median: 4.07 months) than in the PD–PD group (median 8.77 months). Recent studies reported a local control rate of 87–93% at 6 months and 49–91% at 12 months in small-to-medium (1–3 cm)-sized BM lesions treated with 18 Gy^21,30^. According to our results, early histologic local progression may have been overlooked in some patients due to the radiographic RN diagnosis. This would lead to a lower 6-month local control rate.

Surgical debulking was feasible to relieve the neurologic symptoms and improve the performance status in patients with steroid-refractory RN. However, during the median follow-up period of 27.7 months, metastatic progression was observed in 36% of TRC-RN patients. Therefore, TRC resection alone might be insufficient for local control even with pure necrosis. Close follow-up might be required, and the adjuvant treatment option such as postoperative radiation should be considered.

TRC-RN and TRC-PD exhibited a statistically identical course of local recurrence (p = 1.00). According to the cox regression analysis for local recurrence, the PD–PD group was also identified as a significant risk factor for recurrence. Other local factors such as PIV, prescription dose, and hypofractionation did not show significant association with postoperative prognosis. Hypofractionated radiosurgery were known to be associated with higher local control with acceptable incidence of radiation necrosis compared to single fractionation in patients with brain metastases^31,32^. However the patients in this study had symptomatic RN which were refractory to medical treatment. It might be thought that the effect of hypofractionation on the clinical course was considerably reduced by resecting the irradiated area.

The overall survival rate of the patients was more related to the presence of disease control at the type of primary lesion rather than regional brain metastases. The fact that the survival rate of the pathologic PD group—TRC-PD and PD–PD—showed a tendency of inferiority to that of TRC-RN is might be related to the status of the primary systemic lesion. Although the pathology of irradiated metastatic lesions showing TRC was a viable tumor cell, there was no significant difference in clinical course from TRC showing the pathology of pure RN. These results show that the pathological heterogeneity did not significantly affect at least the local prognosis of the tumor. A larger number of multicenter studies is needed to examine the effect of TRC on overall survival in more detail.

This study has some limitations. It is a retrospective single-center study with a small cohort. In the diagnosis of RN, more detailed guidelines for the ratio of necrosis and viable tumor cells and other histological findings that can affect the clinical course are needed. We did not include systemic therapies such as chemotherapy and immunotherapy, possible risk factors for RN, due to the heterogeneity of individual therapeutic regimens.

In conclusion, the histology of symptomatic TRC generated in brain metastases treated with GKS often contained a mixture of viable tumor cells rather than pure necrosis. Despite this heterogeneity, there was no significant difference in the postoperative clinical course of local lesions with TRC features. Although the pathology was progressive disease, it was confirmed that the group with TRC had a better local prognosis than the group with PD in advanced MRI diagnosis. Also, even in the pure necrotic lesion, there is a possibility of recurrence in long-term follow-up, thus regular follow-up and adjuvant therapy might be needed.

## Material and methods

### Study cohort

The medical records of 201 patients who underwent surgical resection after GKS for BM at our institute between April 2009 and May 2019 were retrospectively reviewed. The Samsung Medical Center institutional review board approved this study and informed consent was waived by the Samsung Medical Center institutional review board due to retrospective analyses (IRB number: 2020-11-147-001). All medical performance in this study were conducted according to current diagnostic or treatment guidelines. Among the patients who Patients with GKS dosimetry reports, advanced MRI sequences to discriminate TRCs and PD, and sufficient clinical follow-ups using MRI after surgery were included. We excluded patients with other surgical pathologies, acute intracranial hemorrhage or abscess, and preoperative GKS as adjuvant therapy.

### Radiosurgical treatment

Radiosurgery was performed with the Leksell Gamma Knife Perfexion from 2009 to 2015 or the Leksell Gamma Knife Icon (both Elekta AB, Stockholm, Sweden) from April 2016 onward. The 1.0-mm slices of T1-weighted and 2.0-mm slices of T2-weighted fluid-attenuated inversion recovery (FLAIR) contrast-enhanced MR images were obtained and transferred to the Leksell GammaPlan Software version 11.1.1 (Elekta AB). Patients with tumor volume larger than 10 cc, maximal tumor diameter > 3 cm and retreatment metastatic lesion were indicated for the hypo-fractionated radiotherapy^33–35^. The gross target volume and clinical target volume were defined identically to the perimeter of T1 enhancement in MRI. The planning target volume or prescription isodose volume (PIV) was created with 0 mm margin in single or two fractionated radiosurgery and with 2 mm margin in three or four fractionated radiosurgery. The dosimetry planning was conducted in accordance with the Radiation Therapy Oncology Group 90–05 study guidelines^36,37^.

Among these indicators, PIV was used in the analysis as a radiosurgical factor. The irradiated volume, rather than the geometric volume of the tumor, has more potential to influence the clinical course of metastatic lesions.

### MRI protocols and image diagnoses

The patients were clinically followed-up and evaluated with MRI every 2–3 months after radiosurgery. Each patient was scanned by 3-T MRI using the brain tumor MRI protocol in our institution, including pre- and post-contrast 5-mm-thick T1-weighted imaging, 5-mm-thick fat-suppressed echo T2-weighted imaging, 5-mm-thick FLAIR imaging, diffusion weighted imaging (DWI), which was performed with spin echo using a b-value of 0 and 1000 s/mm^2^ and dynamic susceptibility contrast (DSC)-perfusion-weighted imaging (PWI) (Dotarem; Guerbet, Aulnay-sous-Bois, France). T2*-perfusion data were processed for the maximum regional cerebral blood volume (rCBV) ratio of non-enhanced (NE) and contrast-enhanced (CE) portions, and rCBV maps were computed using the software provided by the MR vendor (Intellispace Portal 9.0; Philips Health Tech, Best, the Netherlands)^38,39^.

Radiologic diagnoses were made by two experienced neuroradiologists (S.T.K. and J.S.R, both with more than 15-years of neuroradiologic practice). For post-GKS lesions that exhibited increased T1-enhancement with T2/FLAIR hyperintensity suggesting the volumetric mass effect in follow-up MRI, tumor progression was suspected if a solid enhancement pattern, higher rCBV, and more restricted diffusion were observed on qualitative inspection. TRCs were preferably diagnosed based on the “soap bubble” or “Swiss cheese” sign of peripheral enhancement, centrally necrotic lesions on T1-weighted post-contrast sequence, low or constant rCBV, and less restricted diffusion^6,7,10,22,24^.

### Indication for surgical resection and histopathologic diagnosis

For patients who presented post-GKS neurological deficits or symptoms of increased intracranial pressure (IICP), treatment plan was thoroughly discussed on the Neuro-oncology conference. Symptomatic patients with imaging diagnoses of RN or PD were primarily treated with oral steroids. Then we conducted surgical resection of metastatic lesions if neurologic symptoms are refractory for medical treatment and patient’s conditions are tolerable to surgery, which Karnofsky performance scale (KPS) score is larger than 60 and life expectancy longer than 3 months.

Based on histopathological diagnosis, patients were classified into the following groups: PD group when their resected specimen showed prominent tumor cells (> 30% of the lesion composed of tumor cells); mixed PD/RN group when their resected specimen showed predominant radiation-induced brain necrosis (> 70% of the lesion) with viable tumor cells (< 30% of the lesion); and RN group when no viable tumor cells were found in their specimens.

Patients underwent postoperative MRI within 48 h and routine outpatient follow-up 1 month after surgery and were further evaluated at 3-month intervals thereafter, and data were reported up to November 2020.

### Statistical analyses

Patient demographics and clinical data were summarized using standard descriptive statistics and frequency tabulation. If statistically significant results indicated non-equal distribution, post hoc tests were used to identify significant differences between the groups; Bonferroni correction method for the median values of continuous variables and Jonckheere-Terpstra test for categorical variables. OS of patients was calculated from the time of resection until death or last follow-up. PFS was defined as the time from the surgical resection to the diagnosis of local progression of the resected lesion. Survival probability was estimated using the Kaplan–Meier product-limit method. To identify the risk factors for local progression and death, we used the Cox proportional-hazards model. Clinical variables were included in multivariable cox regression if they were significantly different between three groups or the 95% confidence interval of HR did not cross 1 with p-value < 0.1 in univariable Cox regression analyses. Statistical significance was determined when the p-value was < 0.05. SAS version 9.4 (SAS Institute, Cary, NC) was used for all statistical analyses.

## Supplementary Information

Supplementary Information.
