# Supplementary material for: Histopathology and surgical outcome of symptomatic treatment-related changes after gamma knife radiosurgery in patients with brain metastases
Source: Sci Rep. 2022 Feb 22;12:3013. doi: 10.1038/s41598-022-06881-6 (PMC8863779; doi:10.1038/s41598-022-06881-6)
Supplement: Supplementary file 1 — Supplementary Information. [file 41598_2022_6881_MOESM1_ESM.pdf]

Samsung Medical Center Institutional Review Board
Panel A

as of Apr 20, 2021

	Name	Earned Degrees	Scientific Status	Representative Capacity	Indications of Experience	Relationship to the SMC	Office on IRB	Gender	Membership Status
1	On, Young Keun	M.D., Ph.D.	Scientist	Cognitively impaired adults/Adults unable to consent/Elders	Professor of Cardiology, SKKU School of Med, and SMCIRB member since Mar 9, 2010	Current employee	IRB Secretary/ Chair of Panel A	M	Member
2	Choi, Hyun Il	M.D.	Scientist	Pregnant women/ Children & Minors	Gyenecologist, Superintendent of SAM Women's Hospital, and SMC IRB member since Mar 9, 2010	External member	Vice Chair of Panel A	M	Member
3	Park, Chaehwa	Ph.D.	Scientist	Pregnant women/ Children & Minors	Biologist at Basic Medical Science Dept, Research Professor of SKKU School of Med, SMC IRB member since Oct 16,	Current employee		F	Member
4	Oh, Dongryul	M.D	Scientist	Cognitively impaired adults/Adults unable to consent/Elders	Associate Professor of Radiation Oncology, SKKU School of Med,and SMC IRB member since Apr 14, 2016	Current employee		M	Member
5	Jeong, Byeong-Ho	M.D., Ph.D.	Scientist	Cognitively impaired adults/Adults unable to consent/Elders	Assistant Professor of Pulmonary and Critical Care Medicine, SKKU School of Med, and SMC IRB member since Mar 2, 2018	Current employee		M	Member
6	Ahn, Hyun Joo	M.D., Ph.D.	Scientist	Cognitively impaired adults/Adults unable to consent/Elders	Professor of Anesthesiology and Pain Medicine, SKKU School of Med, and SMC IRB member since Mar 2, 2019	Current employee		F	Member
7	Kim, Seung Tae	M.D., Ph.D.	Scientist	Cognitively impaired adults/Adults unable to consent/Elders	Associate Professor of Hematology/Oncology, SKKU School of Med, and SMC IRB member since Jul 15, 2016	Current employee		M	Member
8	Kim, Kyung A	Ph.D.	Scientist	Cognitively impaired adults/Adults unable to consent	Research Associate Professor of Statistics and Data Center, and SMC IRB member since Dec 1, 2014	Current employee		F	Member
9	Chung, Seon Young	M.S.	Scientist	Pregnant women/ Children & Minors	Pharmacist at Dept of Pharmacy, and SMC IRB member since Apr 20, 2007	Current employee		F	Member
10	Kwon, In Gak	R.N., Ph.D.	Scientist	Adults unable to consent/Elders	Professor of Clinical Nursing Science, SKKU and SMC IRB member since Jul 16, 2018	Current employee		F	Member
11	Song, Hyo Seok	M.S.W.	Non-scientist	Cognitively impaired adults/Adults unable to consent	Social Worker, Staff at Social contribution Part, and SMC IRB member since Mar 1, 1995	Current employee		M	Member
12	Yoo, Seong Hwa	B.A.	Non-scientist	Pregnant women/ Children & Minors	Working at Art & Company, and SMC IRB member since Jul 1, 2013	External member		F	Member
13	Choi, Cung Hee	LL.B.	Non-scientist	Cognitively impaired adults/Adults unable to consent	Attorney of C&E LLC., and SMC IRB member since Mar 1, 2013	External member		M	Member

Appointment Period: Mar 01, 2021~Feb 28, 2023

Samsung Medical Center Institutional Review Board
Panel B

as of Apr 20, 2021

	Name	Earned Degrees	Scientific Status	Representative Capacity	Indications of Experience	Relationship to the SMC	Office on IRB	Gender	Membership Status
1	Park, Won	M.D., Ph.D.	Scientist	Cognitively impaired adults/Adults unable to consent/Elders	Professor of Radiation Oncology, SKKU School of Med, and SMC IRB member since Apr 14, 2016	Current employee	Chair of Panel B	M	Member
2	Choi, Joon Young	M.D., Ph.D.	Scientist	Cognitively impaired adults/Adults unable to consent/Elders	Professor of Nuclear Medicine, SKKU School of Med, and SMC IRB member since Mar 9, 2010	Current employee	Vice Chair of Panel B	M	Member
3	Yang, Kwang-Mo	M.D., M.S.	Scientist	Adults unable to consent/Elders	Associate Professor of Center for Health Promotion, and SMC IRB member since Mar 2, 2018	Current employee		M	Member
4	Kim, Ji-Yeon	M.D., Ph.D.	Scientist	Cognitively impaired adults/Adults unable to consent/Elders	Assistant Professor of Hematology/Oncology, and SMC IRB member since Mar 2, 2019	Current employee		F	Member
5	Park, Taek Kyu	M.S	Scientist	Adults unable to consent/Elders	Assistant Professor of Cardiology, SKKU School of Med, and SMC IRB member since Jun 17, 2019	Current employee		M	Member
6	Jeong, Han Sin	M.D., Ph.D.	Scientist	Elders/Children & Minors	Professor of Otorhinolaryngology-head and neck surgery, SKKU School of Med, and SMC IRB member since Apr 20,	Current employee		M	Member
7	Park, Hyo Jung	M.S	Scientist	Pregnant women/ Children & Minors	Pharmacist at Clinical pharmacy service Part, and SMC IRB member since May 26, 2014	Current employee		F	Member
8	Cheon, Seoung Min	B.S.	Scientist	Cognitively impaired adults/Adults unable to consent/Elders	Biomedical engineering Part(Main building), and SMC IRB member since Oct 1, 2015	Current employee		M	Member
9	Park, Boram	Ph.D.	Scientist	Pregnant women/ Children & Minors	Biostatistics and Statistics and Data Center, and SMC IRB member since Sep 10, 2020	Current employee		F	Member
10	Kim, Hyun Wook	LL.B.	Non-scientist	Cognitively impaired adults/Adults unable to consent	Attorney of Shin&Kim LLC., and SMC IRB member since Dec 18, 2015	External member		M	Member
11	Cho, Sang Ok	M.A.	Non-scientist	Pregnant women/ Children & Minors	Assistant Professor of Woosong Univ.,and SMC IRB member since Mar 10, 2006	External member		F	Member
12	Han, Ju Hee	B.S	Non-scientist	Pregnant women/ Children & Minors	SMC IRB member since Sep 01, 2017	External member		F	Member

Appointment Period: Mar 01, 2021~Feb 28, 2023

Samsung Medical Center Institutional Review Board
Panel C

as of Apr 20, 2021

	Name	Earned Degrees	Scientific Status	Representative Capacity	Indications of Experience	Relationship to the SMC	Office on IRB	Gender	Membership Status
1	Song, Yoon Mi	M.D., Ph.D.	Scientist	Elders/Children & Minors	Professor of Family Medicine, SKKU School of Med, and SMC IRB member since Mar 1, 2011	Current employee	Chair of Panel C	F	Member
2	Kim, Ki Hyun	M.D., Ph.D.	Scientist	Cognitively impaired adults/Adults unable to consent/Elders	Professor of Hematology/Oncology, SKKU School of Med, and SMC IRB member since Sep 15, 2018	Current employee	Vice Chair of Panel C	M	Member
3	Cho, Kyoung Sam	M.D., Ph.D.	Scientist	Cognitively impaired adults/Adults unable to consent/Elders	Former Professor of Hematology/Oncology, Kyung Hee University Medical Center, and SMC IRB member since Mar 2, 2019	External member		M	Member
4	Song, Hong Ji	M.D., Ph.D.	Scientist	Cognitively impaired adults/Adults unable to consent/Elders	Professor of Family Medicine, Hallym Univ Sacred Heart Hospital, and SMC IRB member since Apr 14, 2016	External member		F	Member
5	Jeon, Kyeong Man	M.D., Ph.D.	Scientist	Cognitively impaired adults/Adults unable to consent/Elders	Professor of Critical Care Medicine, SKKU School of Med, and SMC IRB member since Mar 1, 2013	Current employee		M	Member
6	Shin, Soo Yong	Ph.D.	Scientist	Elders	Assistant Professor of DigitalHealth, SKKU, and SMC IRB member since Jun 17, 2019	Current employee		M	Member
7	Kim, Dong-Wook	Ph.D.	Scientist	Elders	Statistician, Professor of Statistics, SKKU, and SMC IRB member since Jun 17, 2019	External member		M	Member
8	Yu, Su Mi	M.S.	Scientist	Pregnant women/ Children & Minors	Pharmacist, VHS Medical Center, and SMC IRB member since Sep 01, 2017	External member		F	Member
9	Kim, Hyunjin	M.S.	scientist	Pregnant women/ Children & Minors	SMC IRB member since Sep 10, 2020	External member		F	Member
10	Koo, Youngshin	R.N.,LL.B.	Scientist	Pregnant women/ Children & Minors	Attorney of Jehyun LLC, and SMC IRB member since Dec 1, 2018	External member		F	Member
11	Jung, Sung-Hoon	LL.M.	non-Scientist	Elders	Hospital Administrative Staff at R&D planning Team, and SMC IRB member since Apr 1,2012	Current employee		M	Member
12	Cho, Eun-Kyong	M.B.A	Non-scientist	Pregnant women/ Children & Minors	Hospital Administrative Staff at Post Graduate Administration Team, and SMC IRB member since Apr 1,	Current employee		F	Member

Appointment Period: Mar 01, 2021~Feb 28, 2023

Samsung Medical Center Institutional Review Board
Panel D

as of Apr 20, 2021

	Name	Earned Degrees	Scientific Status	Representative Capacity	Indications of Experience	Relationship to the SMC	Office on IRB	Gender	Membership Status
1	Lee, Joo Heung	M.D., Ph.D.	Scientist	Cognitively impaired adults/Children & Minors	Professor of Dermatology, SKKU School of Med, and SMC IRB member since Mar 1, 2005	Current employee	IRB Vice President/ Chair of Panel	M	Member
2	Park, Joon Oh	M.D., Ph.D.	Scientist	Cognitively impaired adults/Adults unable to consent/Elders	Professor of Hematology/Oncology, SKKU School of Med, and SMC IRB member since Sep 15, 2018	Current employee	Vice Chair of Panel D	M	Member
3	Chang, Won Hyuk	M.D., Ph.D.	Scientist	Cognitively impaired adults/Adults unable to consent/Elders	Associate Professor of Physical and Rehabilitation Medicine, SKKU School of Med, and SMC IRB member since Dec 5, 2016	Current employee		M	Member
4	Chun, So Hyun	M.D., M.S.	Scientist	Cognitively impaired adults/Adults unable to consent/Elders	Assistant Professor of International Healthcare Center, and SMC IRB member since Apr 20, 2020	Current employee		F	Member
5	Chung, Ji Eun	Pharm.D, Ph.D.	Scientist	Pregnant women/ Children & Minors	Professor of Clinical Pharmacology, Hanyang Univ., and SMC IRB member since Sep 01, 2017	External member		F	Member
6	Kim, Seon Woo	Ph.D.	Scientist	Pregnant women/ Children & Minors	Biostatistics and Statistics and Data Center, and SMC IRB member since Mar 1, 1997	Current employee		F	Member
7	Suh, Yi-Jong	Ph.D.	Non-scientist	Cognitively impaired adults/Adults unable to consent/Elders	Sociologist, Professor of Sociology, Seoul National Univ, and SMC IRB since Mar 9, 2010	External member		M	Member
8	Yoo, Seong Hwa	B.A.	Non-scientist	Pregnant women/ Children & Minors	Working at Art & Company, and SMC IRB member since Jul 1, 2013	External member		F	Member
9	Shim, Myung-Ah	B.A.	Non-scientist	Pregnant women/ Children & Minors	Hospital Administrative Staff, Director of Dept of MedicalInformation, and SMC IRB member since Aug 1,	Current employee		F	Member
10	Kim, Ae Ran	R.N., Ph.D.	Scientist	Pregnant women/ Children & Minors	Nursing staff Development team, and SMC IRB member since Mar 01, 2021	Current employee		F	Member
11	Kim, Miji	LL.B.	Non-scientist	Cognitively impaired adults/Adults unable to consent	Attorney of I&S LLC., and SMC IRB member since Apr 20, 2020	External member		F	Member

Appointment Period: Mar 01, 2021~Feb 28, 2023

Samsung Medical Center Institutional Review Board
Panel E

as of Apr 20, 2021

	Name	Earned Degrees	Scientific Status	Representative Capacity	Indications of Experience	Relationship to the	Office on IRB	Gender	Membership Status
1	Kim, Jung-ryul	M.D., Ph.D.	Scientist	Cognitively impaired adults/Adults unable to consent/Elders	Associate Professor of Clinical Pharmacology and Therapeutics, and SMC IRB member since Oct 1, 2010	Current employee	Chair of Panel E	M	Member
2	Kim, Hyungjin	M.D., Ph.D.	Scientist	Cognitively impaired adults/Adults unable to consent/Elders	Professor of International health services, SKKU School of Med, and SMC IRB member since Nov 13, 2017	Current employee	Vice Chair of Panel E	M	Member
3	Kim, Hee Taek	Ph.D.	Scientist	Cognitively impaired adults/Adults unable to consent/Elders	Oriental Medical Doctor, Associate Professor of Oriental Medicine, Semyung Univ, and SMC IRB member since Mar 1, 2009	External member		M	Member
4	Lee, SangHoon	M.D.	Scientist	Adults unable to consent/Children & Minors	Associate Professor of Pediatric Surgery, SKKU School of Med, and SMC IRB member since Apr 14, 2016	Current employee		M	Member
5	Cha, Wonchul	M.D., M.S.	Scientist	Cognitively impaired adults/Adults unable to consent/Elders	Assistant Professor of Emergency Medicine, SKKU School of Med, and SMC IRB member since Jul 16, 2018	Current employee		M	Member
6	Kim, Young Hwan	M.D., Ph.D.	scientist	Cognitively impaired adults/Children & Minors	Professor of Nuclear Medicine at Kangbuk Samsung Hospital, SKKU School of Med,and SMC IRB member since Apr 20, 2020	External member		M	Member
7	Park, Mi Ra	Ph.D.	Scientist	Pregnant women/ Children & Minors	Statistician, Professor of Preventive Medicine, Eulji Univ School of Med, and SMC IRB member since Mar 1, 2013	External member		F	Member
8	Chae, Young Han	B.S	Scientist	Cognitively impaired adults/adults unable to consent	Spatial Planning Part, and SMC IRB member since Mar 2, 2018	Current employee		M	Member
9	Lee, Ju Yeun	Ph.D.	Scientist	Pregnant women/ Children & Minors	Pharmacist, Professor of Pharmacy, Seoul National Univ, and SMC IRB member since Mar 1, 2015	External member		F	Member
10	Lee, Jae Sun	RN.	scientist	Pregnant women/ Children & Minors	SMC IRB member since Dec 16, 2019	External member		F	Member
11	Park, Tae Shin	LL.B.	Non-scientist	Cognitively impaired adults/Adults unable to consent	Lawer, Associate Professor of Law School, Chonbuk National Univ., and SMC IRB member since Mar 1, 2011	External member		M	Member
12	Rhee, Youn Jin	B.A.	Non-scientist	Pregnant women/ Children & Minors	Institutional review board Team, and SMC IRB member since Mar 1, 2015	Current employee	IRB Secretary	F	Member

Appointment Period: Mar 01, 2021~Feb 28, 2023

SAMSUNG MEDICAL CENTER

Samsung Medical Center Institutional Review Board
Panel F

(Institutional Bioethics Review Board for Embryo Production and Study)

as of Apr 20, 2021

	Name	Earned Degrees	Scientific Status	Representative Capacity	Indications of Experience	Relationship to the	Office on IRB	Gender	Membership Status
1	Kim, Ho joong	M.D., Ph.D.	Scientist	Pregnant women/ Elders/Children & Minors	Professor of Pulmonary, SKKU School of Med, and SMC IRB member since Mar 8, 2001	Current employee	IRB President/ Chair of Panel F	M	Member
2	Ko, Jae Wook	M.D., Ph.D.	Scientist	Pregnant women/ Children & Minors	Professor of Clinical Pharmacology and Therapeutics, SKKU School of Med, and SMC IRB member since Apr 1, 2019	Current employee		M	Member
3	Kim, Jae Hyeon	M.D., Ph.D.	Scientist	Cognitively impaired adults/Elders/Adults unable to consent	Professor of Endocrinology and metabolism, SKKU School of Med,and SMC IRB member since Dec 16, 2019	Current employee		M	Member
4	Choi, Jae-Hyuck	LL.B.	Non-scientist	Cognitively impaired adults/Adults unable to consent	Attorney, Director of Legal Affairs, and SMC IRB since Jan 15, 2012	Current employee	Vice Chair of Panel F	M	Member
5	Jung, Sunha	RN.	scientist	Cognitively impaired adults/Elders/Adults unable to consent	SMC IRB member since Dec 01, 2018	External member		F	Member
6	Lee, Jae Sun	RN.	scientist	Pregnant women/ Children & Minors	SMC IRB member since Dec 16, 2019	External member		F	Member
7	Lee Min Kyoung	B.A	non-Scientist	Elders	Institutional review board Team, and SMC IRB member since March 1, 2017	Current employee	IRB Secretary	F	Member

Appointment Period: Mar 01, 2021~Feb 28, 2023

Samsung Medical Center Institutional Review Board
Panel G

as of Apr 20, 2021

	Name	Earned Degrees	Scientific Status	Representative Capacity	Indications of Experience	Relationship to the	Office on IRB	Gender	Membership Status
1	Kim, Duck-An	M.D., Ph.D.	Scientist	Pregnant women/ Children & Minors	Professor of Laboratory Medicine, Hanyang Univ School of Med, and SMC IRB member since Mar 1, 2013	External member	Chair of Panel G	M	Member
2	Kim, Sang Jin	M.D., Ph.D.	Scientist	Elders/Children & Minors	Associate Professor of Ophthalmology, SKKU School of Med, and SMC IRB member since Mar 1, 2015	Current employee	Vice Chair of Panel G	M	Member
3	Kye, Seung beom	D.D.S., Ph.D.	Scientist	Cognitively impaired adults/Adults unable to consent/Elders	Professor of Dentistry, SKKU School of Med, and SMC IRB member since Nov 1, 2006	Current employee		M	Member
4	Kim, Tae-Eun	M.D., Ph.D.	Scientist	Pregnant women/ Children & Minors	Clinical Assistant Professor of Dept of Clinical Pharmacology, Konkuk University Medical Center, and SMC IRB member since Jul 1, 2011	External member		F	Member
5	Kim, Hee Jin	M.D., Ph.D.	Scientist	Cognitively impaired adults/Adults unable to consent/Elders	Assistant Professor of Neurology, SKKU School of Med, and SMC IRB member since Mar 2, 2019	Current employee		F	Member
6	Lee, Myung hee	Ph.D.	Scientist	Adults unable to consent/Elders	Social Information Research Institute, and SMC IRB member since Sep 01, 2017	External member		F	Member
7	Park, Hye Ran	M. Pharm.	Scientist	Pregnant women/ Children & Minors	Pharmacist, and SMC IRB since Mar 1, 2011	External member		F	Member
8	Jung, Sun-Woo	LL.B.	Scientist	Cognitively impaired adults/Adults unable to consent	Attorney, Legal Affairs, and SMC IRB member since Apr 1, 2012	Current employee		M	Member
9	Choe, BI	M.B.A., LL.M., Ph.D.	Non-scientist	Cognitively impaired adults/Adults unable to consent	Bioethicist, Associate Professor of Bioethics, The Catholic Ur	External member		M	Member
10	Kim, Ji Sun	M.P.H.	Non-scientist	Pregnant women/ Children & Minors	SMC IRB member since Mar 1, 2013	External member		F	Member

Appointment Period: Mar 01, 2021~Feb 28, 2023

Samsung Medical Center Institutional Review Board
Panel H
(Institutional Bioethics Review Board for Human Biological Specimen Bank)

as of Apr 20, 2021

	Name	Earned Degrees	Scientific Status	Representative Capacity	Indications of Experience	Relationship to the	Office on IRB	Gender	Membership Status
1	Kim, Jong-won	M.D., Ph.D.	Scientist	Pregnant women/ Children & Minors	Professor of Laboratory Medicine, SKKU School of Med, and SMC IRB member since Mar 1, 2003	Current employee	Chair of Panel H	M	Member
2	Kim, Jung-ryul	M.D., Ph.D.	Scientist	Cognitively impaired adults/Adults unable to consent/Elders	Associate Professor of Clinical Pharmacology and Therapeutics, and SMC IRB member since Oct 1, 2010	Current employee	Vice Chair of Panel H	M	Member
3	Park, Hye Ran	M. Pharm.	Scientist	Pregnant women/ Children & Minors	Pharmacist, and SMC IRB since Mar 1, 2011	External member		F	Member
4	Song, Hyo Seok	M.S.W.	Non-scientist	Cognitively impaired adults/Adults unable to consent	Social Worker, Staff at Social contribution Part, and SMC IRB member since Mar 1, 1995	Current employee		M	Member
5	Cho, Eun-Kyong	M.B.A	Non-scientist	Pregnant women/ Children & Minors	Hospital Administrative Staff at Post Graduate Administration Team, and SMC IRB member since Apr 1,	Current employee		F	Member
6	Jung, Sunha	RN.	scientist	Cognitively impaired adults/Elders/Adults unable to consent	SMC IRB member since Dec 01, 2018	External member		F	Member

Appointment Period: Mar 01, 2021~Feb 28, 2023

Samsung Medical Center Institutional Review Board
Panel I

SAMSUNG MEDICAL CENTER

as of Apr 20, 2021

	Name	Earned Degrees	Scientific Status	Representative Capacity	Indications of Experience	Relationship to the	Office on IRB	Gender	Membership Status
1	Ahn, Jin Seok	M.D., Ph.D.	Scientist	Cognitively impaired adults/Adults unable to consent/Elders	Professor of Hematology/Oncology, SKKU School of Med, and SMC IRB member since Sep 15, 2018	Current employee	Chair of Panel I	M	Member
2	Jung, Hyun Ae	M.D., Ph.D.	Scientist	Cognitively impaired adults/Adults unable to consent/Elders	Clinical Fellow of Hematology/Oncology, and SMC IRB member since Sep 15, 2018	Current employee		F	Member
3	Ryu, Jai Min	M.D., M.S.	Scientist	Cognitively impaired adults/Adults unable to consent/Elders	Clinical Fellow of Breast Surgery, and SMC IRB member since Mar 2, 2018	Current employee		M	Member
4	Lee, Sun-Joo	M.D., Ph.D.	Scientist	Cognitively impaired adults/Adults unable to consent/Elders	Professor of Urology, Kyung Hee MedicalCenter, and SMC IRB Member since Oct 1, 2010	External member		M	Member
5	Kim, Eun-Young	Pharm.D., Ph.D.	Scientist	Pregnant women/ Children & Minors	Pharmacist, Associate Professor of Pharmacy, Chungang Univ, and SMC IRB member since Jul 20, 2010	External member		F	Member
6	Lee, Heeyoung	Pharm.D., Ph.D.	scientist	Pregnant women/ Children & Minors	Assistant Professor of Clinical Medicinal Sciences, Konyang Univ., and SMC IRB member since Apr 20, 2020	External member		F	Member
7	Kwon, Da-hee	M.B.P	Non-scientist	Pregnant women/ Children & Minors	SMC IRB member since July 16, 2018	External member		F	Member

Appointment Period: Mar 01, 2021~Feb 28, 2023

SAMSUNG MEDICAL CENTER

Samsung Medical Center Institutional Review Board
Panel J

as of Apr 20, 2021

	Name	Earned Degrees	Scientific Status	Representative Capacity	Indications of Experience	Relationship to the	Office on IRB	Gender	Membership Status
1	Jung, Chul Won	M.D., Ph.D.	Scientist	Cognitively impaired adults/Adults unable to consent/Elders	Professor of Hematology/Oncology, SKKU School of Med, and SMC IRB member since Mar 1, 2002	Current employee	Chair of Panel J	M	Member
2	Kang, Cheol-In	M.D., Ph.D.	Scientist	Cognitively impaired adults/Adults unable to consent/Elders	Professor of Infectious Diseases, SKKU School of Med, and SMC IRB member since Mar 1, 2009	Current employee	Vice Chair of Panel J	M	Member
3	Jang, Hye Ryoun	M.D., Ph.D.	Scientist	Pregnant women/ Children & Minors	Associate Professor of Nephrology, SKKU School of Med, and SMC IRB member since Mar 1, 2013	Current employee		F	Member
4	Park, So Jin	M.S.	Scientist	Pregnant women/Children & Minors	Pharmacist at Clinical pharmacy service Part, and SMC IRB member since Mar 2, 2018	Current employee		F	Member
5	Kim, Ji Sun	M.P.H.	Non-scientist	Pregnant women/ Children & Minors	SMC IRB member since Mar 1, 2013	External member		F	Member
6	Lee, Hye Jin	B.A.	Non-scientist	Elders	Institutional review board Team, and SMC IRB member since March 1, 2015	External member	IRB Secretary	F	Member

Appointment Period: Mar 01, 2021~Feb 28, 2023

Samsung Medical Center Institutional Review Board
Panel K

as of Apr 20, 2021

	Name	Earned Degrees	Scientific Status	Representative Capacity	Indications of Experience	Relationship to the	Office on IRB	Gender	Membership Status
1	Song, Sang Yong	M.D., Ph.D.	Scientist	Pregnant women/ Cognitively impaired adults/Adults unable to consent	Professor of Pathology, SKKU School of Med, and SMC IRB member since Oct 15, 2013	Current employee	Chair of Panel K	M	Member
2	Choi, Yoon-La	M.D., Ph.D.	Scientist	Pregnant women/ Children & Minors	Professor of Pathology, SKKU School of Med,and SMC IRB member since Mar 1, 2009	Current employee	Vice Chair of Panel K	F	Member
3	Park, Hyung Doo	M.D., Ph.D.	Scientist	Cognitively impaired adults/Adults unable to consent/Elders	Professor of Laboratory Medicine, SKKU School of Med, and SMC IRB member since Nov 20, 2020	Current employee		M	Member
4	Cho, Duck	M.D., Ph.D.	Scientist	Cognitively impaired adults/Adults unable to consent/Children & Minors	Professor of Laboratory Medicine, SKKU School of Med, and SMC IRB member since Nov 20, 2020	Current employee		M	Member
5	Choi, Saes Byeol	B.S.	Scientist	Pregnant women/ Children & Minors	Institutional review board Team, and SMC IRB member since Nov 20, 2020	Current employee		F	Member
6	Kim, Ji Sun	M.P.H.	Non-scientist	Pregnant women/ Children & Minors	SMC IRB member since Mar 1, 2013	External member		F	Member
7	Jang, Se il	LL.M.MBA	non-Scientist	Elders	Institutional review board Team, and SMC IRB member since Mar 1, 2017	Current employee	IRB Secretary	M	Member

Appointment Period: Mar 01, 2021~Feb 28, 2023

Samsung Medical Center Institutional Review Board Members

IRB Panel is not designated.

as of Apr 20, 2021

	Name	Earned Degrees	Scientific Status	Representative Capacity	Indications of Experience	Relationship to the	Office on IRB	Gender	Membership Status
1	Cho, Hyun In	B.S.	Scientist	Cognitively impaired adults/Adults unable to consent/Elders	Pharmacist, Director of IRB Office, and SMC IRB since Mar 1, 2005	Current employee	IRB Secretary	M	Member
2	Lee, Jeong Eon	M.D., Ph.D.	Scientist	Pregnant women/ Children & Minors	Professor of Breast and Endocrine Surgery, SKKU School of Med, and SMC IRB member since Mar 9,2010	Current employee		M	Member
3	GWAK, Mi Sook	M.D., Ph.D.	Scientist	Pregnant women/ Children & Minors	Professor of Anesthesiology and Pain Medicine, and SMC IRB member since Mar 1, 2011	Current employee		F	Member
4	Seo, Woo-Keun	M.D., Ph.D.	Scientist	Cognitively impaired adults/Adults unable to consent	Professor of Neurology, SKKU School of Med, and SMC IRB member since Dec 5, 2016	Current employee		M	Member
5	Kang, Mi Ra	M.D., M.S.	Scientist	Pregnant women/ Children & Minors	Professor of Center for Health Promotion, and SMC IRB member since Mar 1, 2013	Current employee		F	Member
6	Lee, Suk-Koo	M.D., Ph.D.	Scientist	Adults unable to consent/Children & Minors	Former Professor of Pediatric Surgery, and SMC IRB member since Jan 1, 1999	External member		M	Member
7	Kim, Jong Man	M.D., Ph.D.	Scientist	Cognitively impaired adults/Adults unable to consent/Elders	Associate Professor of Transplantion Surgery, SKKU School of Med, and SMC IRB member since Sep 10, 2020	Current employee		M	Member
8	Kang, Won Seok	M.D., Ph.D.	Scientist	Cognitively impaired adults/Children & Minors	Assistant Professor of Gastroenterology, SKKU School of Med, and SMC IRB member since Mar 01, 2021	Current employee		M	Member
9	Kwon, Jung Hye	M.D., Ph.D.	Scientist	Pregnant women/ Elders/Children & Minors	Associate Professor of Hematooncology Kangdong Sacred Heart Hospital, Hallym Univ, and SMC IRB member since Dec 15, 2015	External member		F	Member
10	Shin, Myung-Hee	M.D., Ph.D.	Scientist	Pregnant women/ Children & Minors	Professor of Preventive Medicine, SKKU School of Med, and SMC IRB member since Mar 1, 2007	Current employee		F	Member
11	Cho, Hee Yeon	M.D., Ph.D.	Scientist	Pregnant women/ Children & Minors	Associate Professor of Pediatrics, SKKU School of Med, and SMC IRB member since Mar 2, 2019	Current employee		F	Member
1	Huh, Woo seong	M.D., Ph.D.	Scientist	Cognitively impaired adults/Adults unable to consent/Elders	Professor of Nephrology, SKKU School of Med, SMC IRB member since Jan 1, 1999	Current employee	IRB Secretary/ Chair of Panel G	M	Member
	Kim, Ji Woon	M.D.	Scientist	Cognitively impaired adults/Adults unable to consent/Elders	Clinical Associate Professor of International Healthcare Center, and SMC IRB member since Apr 20, 2021	Current employee		M	Member
12	Chung, Yujin	Ph.D.	Scientist	Pregnant women/ Children & Minors	Assistant Professor of Applied Statistics, Kyonggi Univ. and SMC IRB member since May 25, 2020	External member		F	Member
13	Jang, Shin Yi	Ph.D.	Scientist	Pregnant women/ Children & Minors	Cardio-vascular imaging Center, and SMC IRB member since May 25, 2020	Current employee		F	Member
14	Jung, Wonhee	M.P.H.	Scientist	Pregnant women/ Children & Minors	Pharmacist, and SMC IRB since Mar 1, 2007	External member	IRB Secretary	F	Member
15	Jun, Hye Won	B.S.N	scientist	Pregnant women/ Children & Minors	Institutional review board Team, and SMC IRB member since Dec 15, 2015	Current employee	IRB Secretary	F	Member
16	Kang, Ju Yeon	B.S.	scientist	Pregnant women/ Children & Minors	Institutional review board Team, and SMC IRB member since March 1, 2015	Current employee	IRB Secretary	F	Member
17	Hwang, Moon Sook	R.N., Ph.D.	Scientist	Elders/Adults unable to consent	Professor of Nursing, Woosuk Univ and SMC IRB member since Oct 16, 2009	External member		F	Member
18	Yoo, Hae Ryong	M.Div., D.Min.	Non-scientist	Cognitively impaired adults/Elders	Dispathced Minister of Somang Presbyterian Church, and SMC IRB member since Apr 10, 2008	External member		M	Member
19	Hong, Soon Hwa	D.Min.	Non-scientist	Cognitively impaired adults/Elders	Minister of Jusim Presbyterian Church, and SMC IRB member since Jul 1, 2004	External member		M	Member
20	Chang, Hee Sook	M.A.	Non-scientist	Pregnant women/ Children & Minors	Musician, and SMC IRB member since Jul 1, 2011	External member		F	Member
21	Jin, Moon Ho	B.A.	Non-scientist	Elders/Adults unable to consent	SMC IRB member since Mar 10, 2008	External member		M	Member
22	Park, Kwan Woo	PharmD, JD	Scientist	Cognitively impaired adults/Adults unable to consent	Attorney of Kim & Chang, and SMC IRB member since Mar 2, 2019	External member		M	Member
22	Kim, Jung Mee	Pharm.D, Ph.D.	Scientist	Pregnant women/ Children & Minors	SMC IRB member since Apr 20, 2021	External member		F	Member

Appointment Period: Mar 01, 2021~Feb 28, 2023

※ Alternate Member

1. All Alternate Member is the active SMC IRB Member.
2. Scientific Member can replace Scientific Member.
3. Non-scientific member can replace Non-scientific Member.
4. But, item 2 & 3 are not necessarily applicable if at least one External Member, at least one Scientific Member and at least one Non-scientific Member can attend the convened meeting.
